# What Is Gynecology?

**Published:** 1892-07

**Authors:** R. R. Kime

**Affiliations:** Lecturer on Gynecology, Southern Medical College, Atlanta, Ga.


### WHAT IS GYNECOLOGY?*

BY

R. R. KIME, M. D.,

Lecturer on Gynecology, Southern Medical College,

ATLANTA, GA.

At first thought it would seem only idle curiosity would prompt such a question. Yet from a more careful consideration it presents an enigma difficult of solution. If we take a retrospective view and look away back into the dim vista of the past, we learn that Hippocrates, B. C. 450, "advised fumigations in amenorrhœa and as a test of fertility. The woman who did not conceive was wrapped in blankets and fumigated from beneath; if scent passed through her body to the nostrils and mouth, then it was known that she was not unfruitful."

He also "maintained that the movement of the uterus toward the head caused pain under the eyes and nose, with abundant and frothy saliva; if it moved toward the hypogastrium, it caused vomiting of an acid, burning matter; if it moved toward the liver, caused loss of speech, clenching of the teeth and a livid skin." It is said the "Father of Medicine" recognized the sympathy existing between the mammary gland and uterus, which he utilized by cupping them for uterine hemorrhage. That molar pregnancy in the unmarried impeached her virtue as it was due to a superabundance of the menstrual blood, with a bad condition of the semen. That he recognized cancer of the uterus, with gloomy prognosis, treated stenosis of the os with applications of verdigris and dealt at length with sterility.

*Read before the Georgia Medical Association, April 21, 1892.

Coming along down the line about twelve centuries we find Aetius, in his writings, speaks of the speculum sponge tent, peri-uterine cellulitis, medicated pessaries, vaginal injections, caustics for ulcers of the os, a sound for replacing the uterus, etc. It is also said that vaginal injections were used as extensively by the Greeks as they are to-day.

Another twelve centuries brings us to the nineteenth century, in which fads, fashions and cycles hold sway as in olden times.

To illustrate we have only to mention that a few years since everybody slit or divided the cervix; a few years later everybody sewed it up; now everbody divulses or dilates and drains. A few years ago everybody probed and sounded the uterus, applied caustics to ulcers of the os ; now but few use the sound, and ulcers of the os are a *nonentity.*

Yesterday was the mechanical age; then every ill was due to displacement of the uterus, and the gynecologist felt it his sacred duty to invent a pessary to hold the uterus into the position that suited his idea of the normal; now it is known that the uterus has no fixed position. So we might continue enumerating one change after another, but this is sufficient to illustrate the point.

For fear that some of the professional brethren may conclude that gynecologists are the only cranks in the profession, I will simply state that none of the gynecologists used Bergeon's "gas treatment" for tubercular salpingitis, nor Brown-Sequard's Elixir for female diseases, except in the good old-fashioned way.

To return to our subject ; what is gynecology? We conclude it can only be answered approximately. Gynecology in the past seems to have had the fiat of change stamped upon it, and so it will be in the future, until it is reduced to an exact science.

Within the last three or four decades, the advancement in gynecology has been so rapid that it is difficult to keep in the line of march as the procession advances to new thoughts and new discoveries. It was within the present century that McDowell first performed ovariotomy (1809), which was an effulgent ray of light preceding a brighter day for suffering woman.

He performed the operation thirteen times with eight recoveries, which record was not equaled by any American operator until the time of Dr. Washington L. Atlee, who reported in 1855 thirty cases with seventeen recoveries. To-day the mortality of expert operators should not exceed ten to twelve per cent. Ovariotomy is about the only gynecological operation that has not been greatly abused or oft resorted to without just provocation.

An improved technique with lessened mortality is about the only improvement since the first operation was performed.

With other diseased conditions of the female genitalia, we find the changes more marked with greater improvements in operations and treatment.

A short time since, minor surgical gynecology was in the ascendancy and fashion; to-day abdominal surgery ranks first. This change has been wrought by the introduction of the principles of aseptic surgery, rendering abdominal sections safe, efficient and practical.

Pozzi says: "The present use of vaginal hysterectomy, ovariotomy, curetting, even shortening of the round ligaments, is merely a revival due to the introduction of antiseptics."

As a result of this change, a new uterine pathology has been developed. To-day true pelvic cellulitis and pelvic hæmatocele are considered rare; the former has been supplanted by pelvic peritonitis, the latter by extra-uterine pregnancy. A great many cases that were formerly considered due to displacements of uterus, lacerated cervices and pelvic cellulitis, are now considered due to disease of tubes or ovaries and consequent results. The latest fashion being to exclude pelvic cellulitis entirely, except as a result of puerperal infection, and regard all such inflammations as pelvic peritonitis a result of pyosalpinx or extension of inflammation from ovaries, tubes or uterus.

The opium plan of treating peritonitis has reached its zenith, and is being overpowered by the exosmotic eliminative action of salines with more satisfactory results.

Oöphorectomy was introduced by a member of this society, to whom we are proud and glad to pay tribute. By others the operation has been greatly abused, and many an ovary has been sacrificed needlessly. The reckless use of the operation is now discountenanced by the profession, while its legitimate sphere remains yet to be established, especially when resorted to for impaired mental conditions.

A gynecologist has well said, "A woman has other organs besides her generative organs," a fact which should be well considered and the diseased condition traced from cause to effect before unsexing a woman for mental derangement. Simply from the fact that a woman's mental faculties are impaired or menstrual functions deranged, is no positive indication for operation ; as well might we conclude that a man should be castrated for mental derangement accompanied by some abnormal condition of generative organs.

In a few properly selected cases the operation is the only thing that will relieve, but the operation should not be performed indiscriminately.

Tait, whose boldness as an operator is equaled by few, has taken up that which was foreshadowed by oöphorectomy and given us some advances in pelvic surgery of material benefit in establishing the utility of certain procedures and demonstrating the frequency of extra-uterine pregnancy. His triumphs were heralded from shore to shore, and removal of the uterine appendages became a very common gynecological procedure and many a woman was unsexed needlessly, but to-day conservatism is being displayed, and the operation is now advised for positive indications only, by gynecologists, for such as pyosalpinx, to check growth of fibroid tumors, removal of tubal pregnancies and for diseased conditions of the appendages themselves.

HYSTERECTOMY, VAGINAL AND SUPRA-VAGINAL, in cancerous conditions, is yet an unsettled question, some eminent authorities recommending high amputation of the cervix in its stead, where the cancerous condition is confined to the cervix.

As the techniqe improves, with a lower death rate, it is probable that vaginal or total abdominal hysterectomy will take precedence.

Fibroids have been treated variously, each plan having its advocates.

Electricity, championed by Apostoli, held sway for a time and promised much, but has failed to maintain its foothold and accomplish what it was hoped it would. Electricity has a good effect in some cases, but at best is only symptomatic and palliative in relief, not devoid of danger, probably causing the death of as many as it has permanently cured.

Removal of the appendages accomplishes the same results as the menopause, and relieves several properly selected cases, while a few remain for hysterectomy as the only hope of relief, which operation is now receiving favor among gynecologists. So far, the extraperitoneal treatment of the pedicle gives the lowest death rate (four per cent. less), although it is not the ideal surgical operation.

In minor gynecology we find a more unsettled, changeable condition, with, at present, a revival of dilatation of the os, now practiced by Goodell; dilatation and drainage, Wylie; dilatation and packing with gauze, Polk. Dysmenorrhœa and its cogeners are no longer considered as diseases, but as symptoms or indications of diseased conditions.

Displacements are no longer relegated to fixed supports without regard to cause or condition, but are treated by means adapted to the individual condition, removing if possible the cause.

Among methods practiced may be found Alexander's operation, hysteropexia; Dudley's operation (*American Journal Obstetrics*, February, '91); Reed's, of Cincinnati (*American Journal Obstetrics*, January, '92); divulsion of cervix, Goodell's; dilatation, curetting, drainage, Wylie; treatment for reduction of enlarged uterus, and last but not least, tamponade of vagina introduced by Taliaferro's patient in knee chest or Sims' position, after reposition of uterus, which also accomplishes in a great measure the same results as the inclined decubitus of Emmet.

Brandt and others have used massage for uterine displacements with and without adhesions, which it is to be hoped will soon fall into disuse, not only on account of the dangers connected with it, but for the sake of morality and for fear some genito-urinary surgeon advises massage of the penis for inflammatory deposits in the urethra or pathological congestions of this organ.

Ulcerations of the os disappeared as if by magic when Emmet brought the torn lips of the cervix together, while diseases of the uterine appendages curtail the use of Emmet's operation, even in some cases rendering it a very dangerous procedure.

Operations for vaginal fistulæ have improved but little since Sims and Jobert de Lamballe established their utility, unless it be Tait's flap splitting method.

For injuries to the perineum and pelvic fascia quite a number of operations have been devised. For injury to the pelvic fascia and lacerations in vaginal sulci Emmet's operation fulfills the indications, while with a central tear, with or without a rectocele, a Hegar or some of its modifications meet most all indications. A superficial rent of perineum or one complete involving recto-vaginal septum to a limited extent is best met by Tait's flap splitting methods, and used by many with gratifying success.

It is a difficult matter to present such a subject as the title of this paper in an intelligent manner in one short article. It has not been the object of the writer to present anything new or to specially enlighten, but simply to direct attention and thought to the changeableness and development of this branch of medical science and refute the oft-repeated expression "all a doctor needs for treating diseases of women is a speculum and sound, with a few applications and tampons." I am glad to say gynecology is fast approaching a scientific basis. In the last two decades it has outstripped all other specialties of medicine in new discoveries and improved operative *technique*, thereby saving many of the lives of—

The noblest of God's creation—a woman perfected.

"The first clinic in diseases of women held in this country was established in 1841 by Dr. Gunning S. Bedford, in University Medical College, New York," and now no medical college can afford to be without a professor of gynecology, or at least a lecturer on that specialty and clinics. It has also been demonstrated that gynecology cannot be successfully taught without practical examination of patient by student, under competent instruction, with bedside observation in operative work. Any medical college that fails to provide such instructions in its curriculum is derelict of its duty and fails to meet the demands of the day and humanity.

This paper would scarcely be complete without stating that the South has not been asleep; that she has furnished her quota of active, original workers in this specialty; that the Southern Surgical and Gynecological Society is maintained within her borders, and among her sons may be found such names as McDowell, Sims, Taliaferro and Battey, with many other eminent active workers in this specialty.

